# Proton Pump Inhibitor Use and Risk of Gastric Cancer: Current Evidence from Epidemiological Studies and Critical Appraisal

**DOI:** 10.3390/cancers14133052

**Published:** 2022-06-21

**Authors:** Tahmina Nasrin Poly, Ming-Chin Lin, Shabbir Syed-Abdul, Chih-Wei Huang, Hsuan-Chia Yang, Yu-Chuan (Jack) Li

**Affiliations:** 1 Graduate Institute of Biomedical Informatics, College of Medical Science and Technology, Taipei Medical University, Taipei 110301, Taiwan; d610108004@tmu.edu.tw (T.N.P.); arbiter@tmu.edu.tw (M.-C.L.); drshabbir@tmu.edu.tw (S.S.-A.); lovejog@tmu.edu.tw (H.-C.Y.); 2 International Center for Health Information Technology (ICHIT), Taipei Medical University, Taipei 110301, Taiwan; gracehuang@tmu.edu.tw; 3 Research Center of Big Data and Meta-Analysis, Wan Fang Hospital, Taipei Medical University, Taipei 110301, Taiwan; 4 Department of Neurosurgery, Shuang Ho Hospital, Taipei Medical University, Taipei 110301, Taiwan; 5 Department of Dermatology, Wan Fang Hospital, Taipei 116081, Taiwan

**Keywords:** proton pump inhibitors, gastric cancer, stomach neoplasms, epidemiology, meta-analysis

## Abstract

**Simple Summary:**

Proton pump inhibitors (PPIs) are prescribed for reducing the amount of stomach acid. PPIs increase the systemic level of gastrin, a trophic hormone, which is reported to be linked with carcinogenesis. Previous epidemiological studies have shown that the use of PPIs increase the risk of gastric cancer, questioning the safety of PPI therapy for reducing gastric acid suppression. The findings of this study show that PPI use is associated with the risk of gastric cancer (RR 1.80, 95% CI, 1.46–2.22, *p* < 0.001) compared with non-users. The evidence from this study suggests that clinicians should maintain heightened vigilance regarding the adverse effect of PPI therapy and weigh the long-term outcomes.

**Abstract:**

Proton pump inhibitors (PPIs) are used for maintaining or improving gastric problems. Evidence from observational studies indicates that PPI therapy is associated with an increased risk of gastric cancer. However, the evidence for PPIs increasing the risk of gastric cancer is still being debated. Therefore, we aimed to investigate whether long-term PPI use is associated with an increased risk of gastric cancer. We systematically searched the relevant literature in electronic databases, including PubMed, EMBASE, Scopus, and Web of Science. The search and collection of eligible studies was between 1 January 2000 and 1 July 2021. Two independent authors were responsible for the study selection process, and they considered only observational studies that compared the risk of gastric cancer with PPI treatment. We extracted relevant information from selected studies, and assessed the quality using the Newcastle−Ottawa scale (NOS). Finally, we calculated overall risk ratios (RRs) with 95% confidence intervals (CIs) of gastric cancer in the group receiving PPI therapy and the control group. Thirteen observational studies, comprising 10,557 gastric cancer participants, were included. Compared with patients who did not take PPIs, the pooled RR for developing gastric cancer in patients receiving PPIs was 1.80 (95% CI, 1.46–2.22, *p* < 0.001). The overall risk of gastric cancer also increased in patients with gastroesophageal reflux disease (GERD), *H. pylori* treatment, and various adjusted factors. The findings were also consistent across several sensitivity analyses. PPI use is associated with an increased risk of gastric cancer in patients compared with those with no PPI treatment. The findings of this updated study could be used in making clinical decisions between physicians and patients about the initiation and continuation of PPI therapy, especially in patients at high risk of gastric cancer. Additionally, large randomized controlled trials are needed to determine whether PPIs are associated with a higher risk of gastric cancer.

## 1. Introduction

Proton pump inhibitors (PPIs) are prescribed for reducing the amount of stomach acid [1]. A growing body of evidence from epidemiological studies indicates that PPIs are associated with reliving gastroesophageal reflux disease (GERD)-related complications, namely oesophageal ulcers, oesophageal bleeding, and peptic stricture [2]. Therefore, the use of PPIs is considered to be a safe and effective therapy for preventing heartburn and acid-related disorders. Previous evidence has also shown that the use of PPIs is associated with a reduced risk of neoplastic progression Barrett’s oesophagus [3] and is not linked to an increased risk of oesophageal adenocarcinoma [4], colorectal cancer [5], or hepatocellular carcinoma (HCC) [6]. If the long-term use of PPIs was able to reduce the risk of a wide variety of health complications such as cancer, PPIs would be an economical and widely available treatment for acid-related disorders.

However, recent epidemiological studies have revealed that PPIs are associated with an increased risk of gastric cancer [7,8]. They highlighted that PPIs reduce gastric acid production, and consequently accelerate the secretion of gastrin, which is linked to an increase in the risk of gastric cancer. Several animal models have shown that hypergastrinemia causes carcinomas in the gastric corpus. Moreover, previous literature reviews reported a possible association between long-term use of PPI and increased risk of cancer [9], including patients after *H. pylori* eradication [10]. Appropriate interpretation of these reviews is difficult because of the limited evidence, weak inclusion criteria, and insufficient details to distinguish the effects across important characteristics (e.g., region, duration, and adjustment for confounding factors).

A study with updated evidence is an unmet need in order to comprehensively assess the association between PPIs and the risk of gastric cancer. This is clinically important, because estimates of gastric cancer risk remain a cornerstone in formulating health policies to reduce healthcare costs and to improve quality of life. Therefore, we conducted an updated systematic review and meta-analysis of epidemiological studies to investigate the association between PPI use and the risk of gastric cancer. Clarifying the actual magnitude of risk of gastric cancer associated with PPI therapy may have important clinical implications for patients at high risk of gastric cancer.

## 2. Methods

This study was conducted according to the PRISMA (preferred reporting items for systematic reviews and meta-analyses) guidelines [11]. This review is not registered.

### 2.1. Data Sources

We developed a comprehensive search strategy with the help of experts in systematic reviews and meta-analyses, and searched popular electronic databases such as PubMed, Embase, Scopus, and Web of Science between 1 January 2000, and 1 April 2022. Search free-text terms were “proton pump inhibitor” OR “proton pump inhibitors” OR “PPIs” AND “gastric cancer” OR “gastric neoplasm” OR “gastric carcinoma” OR “stomach cancer” OR “stomach neoplasm” OR “stomach carcinoma”. We considered only human studies, and language was restricted to English. Additionally, we screened the reference lists from potential original and review articles to confirm if any potential study was missing.

### 2.2. Eligibility Criteria

Included studies had to fulfil the following criteria: (a) observational studies, (b) participants ≥18 years old, (c) studies that examined the impact of PPI treatment on cancer risk, (d) studies that clearly explained the inclusion and exclusion criteria for PPI users and cancer patients, and (e) studies that provided appropriate information about effect sizes with 95% CI. Studies were excluded if they were not published in English, or were published as reviews, case-reports, congress abstracts, or cross-sectional studies.

### 2.3. Data Extraction

All of the titles and abstracts were screened by two authors (T.N.P. and H.-C.Y.) based on predefined criteria, and a random sample was examined by a third author to confirm reliability. The same two authors then evaluated the full texts against the eligibility criteria. Any disagreement during the screening process was resolved by discussion with the main investigator. Finally, these two authors independently extracted the necessary information from the selected full-text articles. They collected information regarding authors, years of publication, study location, study duration, database, study design, number of participants, percentage of female patients, number of PPI users, number of cancer patients, inclusion and exclusion criteria, effect size, and confounding factors.

### 2.4. Risk of Bias Assessment

We used the Newcastle−Ottawa Scale (NOS) to assess the quality of non-randomized studies, which is suggested by the Cochrane library [12,13]. It assesses non-randomized studies (case-control and cohort) in three domain, namelys: (a) selection of study, (b) comparability, and (c) ascertainment of exposure/outcome. A study is classified into low, moderate, and high quality based on the number of stars. A maximum of one “star” is given for each item in the selection and exposure/outcome categories. A maximum of two “stars” is given for comparability. A total of nine “stars” can be given for the three domains. A study that received nine stars was considered to be at low risk of bias, a study that received seven or eight stars was considered to be at moderate risk, and six or less was considered to be at high risk of bias.

### 2.5. Statistical Analysis

The primary outcome measure was the risk of gastric cancer among individuals with PPI use compared with non-users. The adjusted hazard risks (HRs) or odd ratios (ORs) with 95% CIs were used to calculate the overall risk ratios (RRs). The DerSimonian and Laird random effect model was used to calculate the adjusted pooled RRs with 95% CIs. The I^2^ and Q statistics were used to assess the heterogeneity between studies. The heterogeneity was considered to be very low, low, medium, and high if the I^2^ value was 0~25%, 25–50%, 50~75%, and >75%, respectively [14,15]. We evaluated and presented the publication bias using the funnel plot and the Egger’s regression test [16,17].

We also conducted subgroup analyses to determine viable heterogeneity and to investigate whether the existing confounding and clinical factors were linked to any significant variation of the outcome. The following factors were considered: (a) study design; (b) regional impact; (c) quality of the study; (d) study year; (e) whether the study was adjusted for age, gender and smoking status, alcohol, and GERD; and (f) the various type(s) of gastric cancer in the study. Finally, a forest plot was drawn to visually illustrate the overall pooled effect size. We used two-sided tests to calculate all *p*-values. The effect sizes were considered statistically significant if *p* values were less than 0.05.

## 3. Results

### 3.1. Study Selection

**Figure 1:**
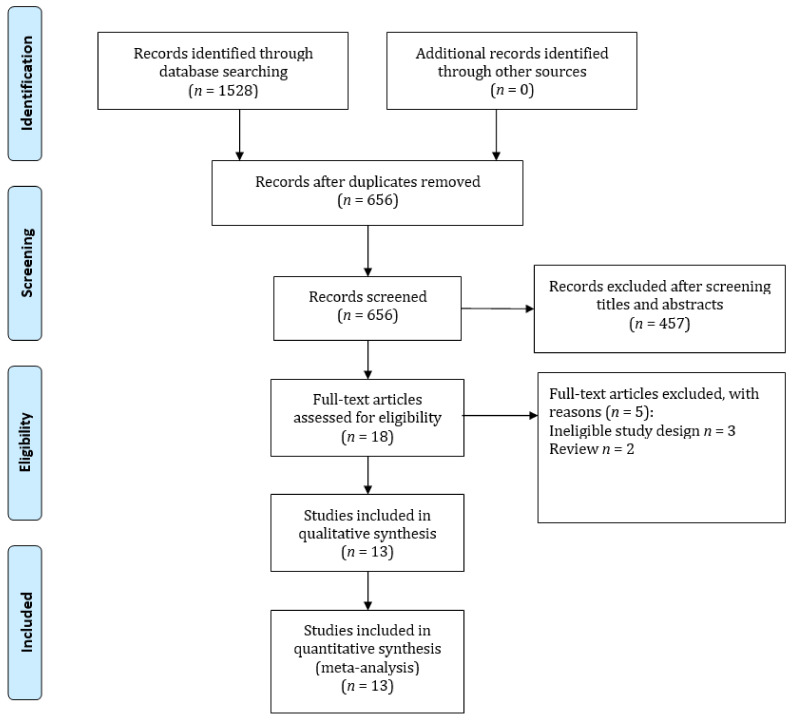
Flow diagram of the literature search and selection of the studies for the meta-analysis.

A total of 1528 studies were identified through our search strategy; 872 articles were removed due to duplications and 656 were went through for further title and abstract evaluation. After assessing 18 full-text articles, 13 articles met all of the inclusion criteria after [7,8,18,19,20,21,22,23,24,25,26,27,28]. Figure 1 shows an overview of the inclusion and exclusion criteria.

### 3.2. Characteristics of the Included Studies

Table 1 presents the baseline characteristics of the included studies. Two of the studies were conducted in North America, five in Europe, and six in Asia. The studies included gastric cancer participants between 21 and 1491 with an age that ranged from 0 and 94 years old. The percentage of female participants was ~58.5%.

### 3.3. Assessment of Risk Bias

The methodological quality of the included studies varied. The quality was high in five studies, moderate in seven studies, and low in one study. The range of the NOS score was between 6 and 9. The average NOS score was 8.07.

### 3.4. PPI Use and the Risk of Gastric Cancer

**Figure 2:**
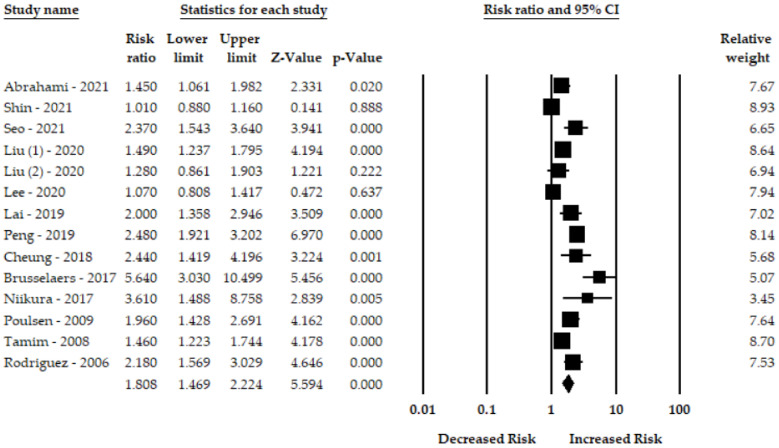
Forest plot of the association between PPIs and risk of gastric cancer [7,8,18,19,20,21,22,23,24,25,26,27,28].

Thirteen studies evaluated the use of PPIs and gastric cancer risk. The pooled risk ratio of gastric cancer for PPI users was 1.80-fold higher (95% CI: 1.46–2.22) than for non-PPIs users. Figure 2 shows that PPI had a significant association gastric cancer. The random-effect model also revealed a significant heterogeneity among the studies (I^2^ = 85.71, Q = 91.03, *p* < 0.001).

### 3.5. Subgroup Analysis

The key target was to make the findings more comprehensive; therefore, we performed robust subgroup analyses. The subgroup analysis was based on the influence of study design, number of participants, region, demographic factors (e.g., adjusted for age and gender), and clinical factors (e.g., GERD and aspirin) (Table 2).

Eight cohort and six case-control studies assessed the association between PPI use and risk of gastric cancer. The pooled RRs for the cohort and case-control studies were 1.99 (95% CI: 1.37–2.88, *p* < 0.001) and 1.69 (95% CI: 1.34–2.13, *p* < 0.001). There was significant heterogeneity among the studies (Q = 58.74, *p* < 0.001, I^2^ = 88.08%, and Q = 25.42, *p* < 0.001, I^2^ = 80.33, respectively).

Six studies from Asia evaluated the impact of PPI treatment on the risk of gastric cancer. The overall pooled RR was 2.07 (95% CI: 1.29–3.30, *p* = 0.02, Q = 58.18, *p* < 0.001, I^2^ = 91.40%). The pooled RRs for studies from Europe and North America were 1.87 (95% CI: 1.41–2.48, *p* < 0.001, number of studies, *n* = 5) and 1.27 (95% CI: 0.94–1.72, *p* = 0.11, *n* = 2), respectively.

The overall pooled RRs for the risk of gastric cancer for studies with ≤ 5000 and >5000 PPI users were 1.74 (95% CI: 1.41–2.14, *p* < 0.001, *n* = 8) and 1.96 (95% CI: 1.20–3.18, *p* = 0.006, *n* = 5), respectively. There was significant heterogeneity among the studies (Q = 32.10, I^2^ = 75.08, *p* < 0.001, and Q = 48.64, I^2^ = 91.77%, *p* < 0.001)

Eight studies adjusted for age when calculating the overall risk of gastric cancer among patients with PPI than that of non-users. The risk of gastric cancer was high, even if adjusted for age (RR: 2.01 95% CI: 1.46–2.75, *p* < 0.001). Moreover, seven studies that adjusted for gender, and four studies adjusted for smoking, as well as alcohol consumption, were also associated with an increased risk of gastric cancer among PPI users. The pooled RRs were 1.94 (95% CI: 1.40–2.69, *p* < 0.001), 1.33 (95% CI: 1.02–1.73, *p* < 0.001), and 1.33 (95% CI: 1.02–1.73, *p* < 0.001), respectively. The overall pooled RRs for the risk of gastric cancer for studies adjusted for gastroesophageal reflux disease (GERD) was 1.84 (95% CI: 1.39–2.44, *p* < 0.001, *n* = 9).

Five studies with a high methodological quality assessed the risk of gastric cancer; the pooled RR was 1.31 (95% CI: 1.06–1.63, *p* = 0.01, *n* = 5), with significant heterogeneity (Q = 19.89, *p* = 0.001, I^2^ = 74.86%). The pooled RRs for moderate and low methodological quality studies were 2.32 (95% CI: 1.74–3.09, *p* < 0.001, *n* = 7) and 2.18 (95% CI: 1.57–3.03, *p* < 0.001, *n* = 1), respectively.

Six studies reported a correlation between PPI therapy and different types of gastric cancer. The use of PPIs was more related to non-cardia gastric cancer (RR: 2.38 95% CI: 1.90–2.98, *p* < 0.001, *n* = 6), while it was less related to cardia gastric cancer (RR: 1.32 95% CI: 0.84–2.03, *p* = 0.21, *n* = 6).

### 3.6. Sensitivity Analysis

We also conducted sensitivity analyses regarding the robustness of this updated study. We excluded two studies (Cheung et al. [22] and Niikura et al. [25]) because both investigated the possible association between long-term use of PPIs and gastric cancer risk after treatment for *H. pylori.* However, no significant change was observed in the pooled effect or heterogeneity (RR 1.72, 95% CI: 1.39–2.13, *p* < 0.001, I^2^ = 86.85).

For dosage of PPIs and medication subtype, Abrahami et al. [28] showed that a higher dose of omeprazole was associated with a higher risk of gastric cancer, compared with a lower dose. The HRs for cumulative omeprazole dose of <14,600 mg, 14,600–28,199 mg, and ≥29,200 mg was 1.33 95% CI: 0.97–1.83 95% CI: 2.051.46–2.89, and 2.34 95% CI: 1.62 to 3.37. Liu et al. [7] also reported that a high dose of PPIs use was associated with an increased risk of gastric cancer (OR:1.23, 95% CI: 0.76–1.97). Patients with omeprazole and lansoprazole also showed a positive association with gastric cancer (HR: 1.17 95% CI: 0.74, 1.85; HR: 1.21 95% CI: 0.71–2.08). When examining PPI intensity of use, Lee at al. [19] showed that the risk of gastric cancer was high when PPIs were taken in the highest dose and for a longer duration (OR: 2.95, 95% CI: 1.23–7.90).

Our findings also showed an increased risk of gastric cancer (RR: 1.98, 95% CI: 1.31–2.98, *p* = 0.001, I^2^ = 51.37, Q = 6.17, *p* = 0.10, *n* = 4) among the patients in the *H. pylori* eradication group. Two studies showed the risk difference between male and female with PPI therapy. The risk of gastric cancer was higher in female patients compared with male patients (RR_male_: 1.28 95% CI: 1.03–1.59, *p* = 0.02, I^2^ = 0, Q = 1.54, *p* = 0.46 and RR_female_: 1.42 95% CI: 0.88–2.29, *p* = 0.14, I^2^ = 79.42, Q = 9.72, *p* = 0.008). Furthermore, the effect of duration on gastric cancer risk was also evaluated. The risk increased with a longer duration (>3 years) of PPI use (2.27 95% CI: 1.11–4.65, *p* = 0.02, *n* = 3).

### 3.7. Publication Bias

**Figure 3:**
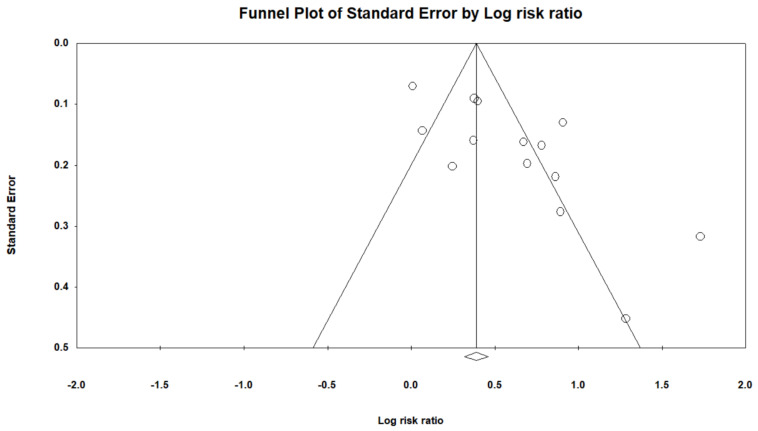
Funnel plot of the risk gastric cancer among patients with PPIs.

We drew a funnel plot to assess publication bias (Figure 3) and the Egger’s test showed evidence of substantial bias (*p* < 0.05).

**Figure 4:**
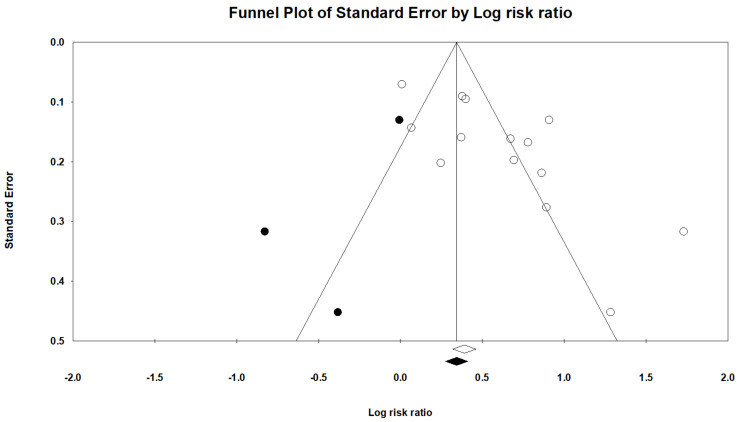
Filled funnel plot of gastric cancer among patients with PPIs.

Therefore, we used the trim-and-fill method, which imputed three studies and generated a symmetrical funnel plot for gastric cancer risk (Figure 4). The RR for gastric cancer was 1.40 (95 % CI, 1.31–1.50; *p* < 0.001) in the trim-and-fill method. However, correction for potential publication bias thus did not change the association between PPIs use and gastric cancer risk.

## 4. Discussion

Using observational studies, this updated meta-analysis assessed the association between PPI use and the risk of gastric cancer, in a bias analysis, and evaluated the robustness of observational associations to unmeasured confounding factors to calculate the possibility of causality. The findings obtained from 13 observational studies show that users of PPIs are at an 80% increased risk of gastric cancer (RR, 1.80 95% CI: 1.46–2.22) compared with non-users. However, when we stratified based on the cumulative duration of PPI use, long-term use (>3 years) of PPIs was also associated with an increased risk of gastric cancer. In the subgroup analyses, the risk increased with the study design, number of participants, adjusted factors (e.g., age and gender), and lag-time. There was significant heterogeneity among studies, which may have originated in the various study designs, definitions of PPI use, gastric cancer identification methods, and different potential confounding factors adjusted. Moreover, included articles were from different regions, covering North America, Europe, and Asia, involving individuals of various ethnicities (e.g., white, black), demographic characteristics, and socioeconomic status. Because of the existence of potential heterogeneity between studies, our findings should be considered with caution. Moreover, the findings from the epidemiological studies (e.g., observational studies) were unable to provide any information whether the possible association was causal or if there were existing confounding factors. Although, for the included observational studies that adjusted for age, gender, smoking and alcohol consumption status, GERD, and aspirin use, the use of PPIs significantly increased the risk of gastric cancer.

In the subgroup analyses, we obtained a lower risk of gastric cancer among the patients with PPIs for high-quality studies than that of moderate- and low-quality studies because of the lack of adjustment of potential confounding factors in these two groups. The findings from adjusted analyses and high-quality studies are more robust, comprehensive, and accurately reflect actual effects. When stratified by region, PPIs showed an increased association with the risk of gastric cancer in all subgroups; however, there was a significantly higher risk of gastric cancer in patients from Asia. The effect of PPIs on gastric cancer can be partially explained by the different races in this region. Previous studies reported the variation of therapeutic response of PPIs because of genetic variability [29,30,31]. Another four subgroup analyses also demonstrated that studies that were adjusted for age, gender, GERD, and/or aspirin [32,33,34], which are considered to be potential confound factors of gastric cancer development, had a higher risk than the unadjusted studies.

The findings of this updated meta-analysis are in line with four previously published systematic review and meta-analyses [10,35,36,37]. Jiang et al. [10] included seven articles with 943,070 patients. The pooled odd ratios show that long-term use of PPIs may possibly increase the risk of gastric cancer (OR 2.50; 95% CI: 1.74–3.85). They were unable to assess publication bias due to the small number of studies, and all studies were retrospective in design. They suggested including more high-quality studies and assessing possible confounding factors to confirm or refute the association. Tran-Duy et al. [35] assessed the effects of PPI therapy on the risks of fundic gland polyps (FGPs) and gastric cancer risk by including 12 studies (eight for FGPs and four for gastric cancer). The findings of their study suggest that PPI therapy is positively associated with an increased risk of gastric cancer. However, the evidence could be biased due to an insufficient amount of studies and unmeasured confounding factors. Wan et al. [36] included seven observational studies, comprising of 926,386 participants. The findings of their study highlighted that long-term use of PPI led to an approximately two-fold increased risk for GC (OR 2.10; 95% CI 1.10–3.09; I^2^ = 97.3%). Finally, Segna et al. [37] conducted a systematic review and meta-analysis, including 13 observational studies with 1,662,881 participants. PPI use had a 1.94-fold higher risk of gastric cancer compared with the non-PPI group. This association should be taken cautiously because the included studies were highly heterogenous. However, our study included a large number of studies and was explicitly designed to evaluate the risk of gastric cancer for PPI users. We assessed the risk of gastric cancer by providing various subgroup analyses (e.g., adjusted for variables such as age and gender) that helped to minimize potential confounding factors. Moreover, we tried to address previous studies’ limitations through carefully selecting studies and through numerous sensitivity analyses.

**Figure 5:**
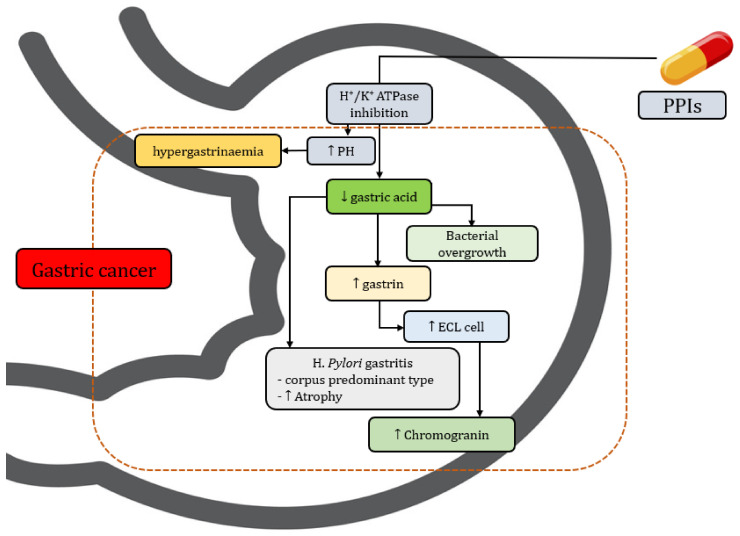
Biological mechanism of PPIs including gastric cancer (↑ = increase, ↓ = decrease).

There are several biological factors that can be used to explain the association between PPI use and gastric cancer (Table 3). The most likely hypothesis for increasing the risk of gastric cancer among patients who use PPIs is hypergastrinaemia [38]. PPIs reduce gastric acid secretion by blocking the H+/K+ ATPase of parietal cells [39], inducing an increase an gastrin secretion from G-cells [40]. Gastrin has long been suspected to be a potential risk factor of gastric cancer by ensuring hypergastrinemia. Previous biological studies also reported a link between hypergastrinemia and gastric tumours [41,42,43,44]. The second hypothesis is that *H. pylori*-related chronic gastritis and atrophy per se have a potential effect on the gastric mucosa and gut microbiota [45,46]. Changing microbial diversity accelerates the progression of gastric cancer [47]. The long-term use of PPIs among patients with *H. pylori* infection may lead to a significant deterioration in gastritis and increased risk of gastric cancer by triggering gastric inflammation and subsequent neoplastic progression (Figure 5). Third, PPIs induce hypoacidity, which increase the production of enterochromaffin-like cells (ECL cells). ECL cells are the key target cells of gastrin in the oxyntic mucosa, and are associated with the expression of cholecystokinin-2 (CCK-2) receptors and the formation of neuroendocrine tumors (NETs) [48].

This updated systematic review with a meta-analysis has several strengths. First, this is the most comprehensive study to date to investigate the risk of gastric cancer among patients with PPI therapy. Given the number of gastric cancer patients from different continents, the broad subgroups, and the sensitivity analyses, this study has immense potential to assess the risk of gastric cancer in patients on PPI treatment. There are several limitations of this study that need to be addressed. First, we were unable to provide information regarding individual PPI (e.g., omeprazole and pantoprazole) use and the risk of gastric cancer. Previous studies showed that short-term treatment with amoxicillin and omeprazole significantly reduced the risk of gastric cancer. Second, it was not possible to stratify the risk of gastric cancer based on dosage, due to a lack of data. As PPIs have proven beneficial effects for the management of the symptoms of several gastric conditions, it is important to clarify the dose and the risk of gastric cancer. Third, the heterogeneity among studies was very high; although this can be explained by the number of studies and the variety of study designs.

## 5. Conclusions

The findings of this updated meta-analysis suggest that the risk of gastric cancer is increased in patients treated with PPIs compared with patients not taking PPIs. The findings are from a summary of observational studies, and PPIs have well-established clinical benefits for patients. Therefore, physicians should regularly assess the actual benefits for patients, especially patients with long-term PPI treatment for GERD. Our study findings also highlight the necessity for long-term randomized controlled trials to evaluate the risk of gastric cancer in patients treated with PPIs.

## Figures and Tables

**Table 1:** Characteristics of the included studies.

Author	Country	Duration	Design	Follow-Up	Total Population	Sex (Female)	Age	PPI	Non-PPI	GC	Log Time (Month)	NOS Score
Abrahami, 2021 [28]	UK	1990–2018	Co	5	1,171,587	45.1	60.4	973,281	198,306	1410	12	9
Shin, 2021 [8]	S. Korea	2004–2015	Co	N/A	77,024	46.6	N/A; >40	39,799	122,118	808	24	9
Seo, 2021 [18]	S. Korea	2002–2013	R-Co	4.3	11,741	52.1	15–94	6877	6877	173	12	8
Liu (1), 2020 [7]	UK	1999–2011	C-C	5.1	6513	42.9	0–70+	1542	4971	1119	12	9
Liu (2), 2020 [7]	UK	1999–2014	Co	4.6	471,779	27.2	0–70+	250	471,323	250	12	9
Lee, 2020 [19]	USA	1996–2016	C-C	N/A	11,776	25.6	72.4 (median)	937	10,839	1233	24	9
Lai, 2019 [20]	Taiwan	2000–2013	C-C	N/A	1298	34.4	65.6 (mean)	539	759	649	12	7
Peng, 2019 [21]	Taiwan	1996–2011	C-C	N/A	2122	N/A	N/A	1693	429	1061	12	8
Cheung, 2018 [22]	Taiwan	2003–2012	R-Co	7.6 (median)	63,397	53.5	54.7	3271	60,126	153	6	9
Brusselaers, 2017 [23]	Sweden	2005–2012	R-Co	4.9	815,700	58.5	N/A; >18	795,490	20,210	1941	12	8
Niikura, 2017 [25]	Japan	1998–2017	R-Co	6.9	533	44	N/A	118	415	21	N/A	7
Poulsen, 2009 [24]	Denmark	1990–2003	R-Co	N/A	36,268	53	40–84	18,790	17,478	161	12	8
Tamim, 2008 [26]	Canada	1994–2003	C-C	N/A	8229	47.9	75.5	1299	6930	1071	6	7
Rodríguez, 2006 [27]	UK	1994–2001	C-C	N/A	10,293	N/A	40–84	442	9786	507	12	6

Note: Co = cohort study; R = retrospective; N/A = not available; GC = gastric cancer; PPI = proton pump inhibitor; NOS = Newcastle−Ottawa scale.

**Table 2:** Subgroup analysis.

Subgroup	No of Study	Effect Size	95% CI	*p*-Value	I^2^	Q-Value	*p*-Value	τ^2^
All studies [7,8,18,19,20,21,22,23,24,25,26,27,28]	13	1.80	1.46–2.22	<0.001	85.71	91.03	<0.001	0.12
Study design								
Cohort [7,8,18,22,23,24,25,28]	8	1.99	1.37–2.88	<0.001	88.08	58.74	<0.001	0.23
Case-control [7,19,20,21,26,27]	6	1.69	1.34–2.13	<0.001	80.33	25.42	<0.001	0.06
Region								
Asia [8,18,20,21,22,25]	6	2.07	1.29–3.30	0.002	91.40	58.18	<0.001	0.28
Europe [7,23,24,27,28]	5	1.87	1.41–2.48	<0.001	77.43	22.16	<0.001	0.09
North America [19,26]	2	1.27	0.94–1.72	0.11	70.27	3.36	0.06	0.03
Methodological quality								
High [7,8,19,22,28]	5	1.31	1.06–1.63	0.01	74.86	19.89	0.001	0.04
Moderate [18,20,21,23,24,25,26]	7	2.32	1.74–3.09	<0.001	78.16	27.47	<0.001	0.10
Low [27]	1	2.18	1.57–3.03	<0.001	-	-	-	-
Adjusted for age								
Yes [7,8,21,22,23,24,25,27]	8	2.01	1.46–2.75	<0.001	89.86	78.91	<0.001	0.18
No [18,19,20,26,28]	5	1.54	1.22–1.95	<0.001	66.98	12.11	0.01	0.04
Adjusted for gender								
Yes [7,8,22,23,24,25,27]	7	1.94	1.40–2.69	<0.001	88.53	61.02	<0.001	0.17
No [18,19,20,21,26,28]	6	1.70	1.30–2.21	<0.001	80.01	25.02	<0.001	0.08
Adjusted for smoking								
Yes [7,8,19,27]	4	1.33	1.02–1.73	0.03	88.65	24.47	<0.001	0.07
No [18,20,21,22,23,24,25,26,28]	9	2.17	1.71–2.76	<0.001	74.37	31.22	<0.001	0.08
Adjusted for alcohol								
Yes	4	1.33	1.02–1.73	0.03	88.65	24.47	<0.001	0.07
No	9	2.17	1.71–2.76	<0.001	74.37	31.22	<0.001	0.08
Adjusted for GERD								
Yes [7,8,18,19,20,21,22,23,27]	9	1.84	1.39–2.44	<0.001	89.25	83.73	<0.001	0.17
No [24,25,26,28]	4	1.66	1.31–2.10	<0.001	51.32	6.16	0.10	0.02
Adjusted for aspirin								
Yes [7,8,18,22,23]	5	1.85	1.26–2.71	0.001	89.80	49.01	<0.001	0.18
No [19,20,21,24,25,26,27,28]	8	1.78	1.42–2.23	<0.001	75.91	29.06	<0.001	0.07
Number of PPI users								
≤5000 [7,19,20,21,22,25,26,27]	8	1.74	1.41–2.14	<0.001	75.08	32.10	<0.001	0.06
>5000 [8,18,23,24,28]	5	1.96	1.20–3.18	0.006	91.77	48.64	<0.001	0.26
Lag time								
<=6 months [22,25,26]	3	1.46	1.03–2.05	0.03	74.25	7.76	0.02	0.06
>6 months [7,8,18,19,20,21,23,24,28]	9	1.86	1.43–2.43	<0.001	88.56	78.67	<0.001	0.15
Cancer type								
Cardia [7,21,22,23,26,27]	6	1.32	0.84–2.03	0.21	58.92	12.17	0.03	0.18
Non-cardia [7,21,22,23,26,27]	6	2.38	1.90–2.98	<0.001	0	4.86	0.43	0

**Table 3:** Summary of biological studies that investigated the association between PPIs and gastric cancer.

Drug	Mechanism	References
PPIs	Gastrin stimulated ECL cell proliferation	[49,50,51]
	Activating the JAK2/STAT3/PI3K/Akt pathway	[52]
	Stimulate the expression of EP2 and EP4 receptors, and upregulate and increase the release of vascular endothelial growth factor	[53]
